# Co-existence of Double Gallbladder and Choledochal Cyst in a Single Patient

**DOI:** 10.7759/cureus.20737

**Published:** 2021-12-27

**Authors:** Abdulaziz A Arishi, Amin Ahmed, Samer Alharthi, Giana Dawod, Wesley Judy, David G Heidt

**Affiliations:** 1 General Surgery, The University of Toledo College of Medicine and Life Sciences, Toledo, USA

**Keywords:** gallbladder disease, type i choledochal cyst, choledochal cyst excision, cholecystectomy, double gallbladder

## Abstract

Additional anatomical structures are rare but can be mistaken for other conditions, causing misdiagnoses and poor outcomes for patients. The presence of concurrent anomalies within the extra structures further complicates a rare situation. We present a case of a patient with two gallbladders and a choledochal cyst diagnosed via radiography and confirmed by exploratory laparotomy. He underwent a cholecystectomy, choledochal cyst resection, and hepaticojejunostomy, and he was doing well as of his last follow-up. This case highlights the need to consider radiological imaging in patients with choledochal cysts carefully.

## Introduction

Knowledge of anatomy and a high level of alertness for congenital abnormalities is critical for safe surgical operations. A double gallbladder is a rare anomaly of biliary disease, with an incidence rate of one in 3,000 to 4,000 cases [1]. A double gallbladder can be mistaken for diverticulum gallbladder, adenomyosis, gallstones, pericholecystic fluid, or choledochal cysts. Choledochal cysts are a rare dilation of the bile duct, and incidence ranges from one in 13,000 to 200,000 patients [2]. Patients with choledochal cysts are at an increased risk of cholangitis, pancreatitis, and malignancy. We present the first reported case with two gallbladders and a choledochal cyst.

## Case presentation

**Figure 1:**
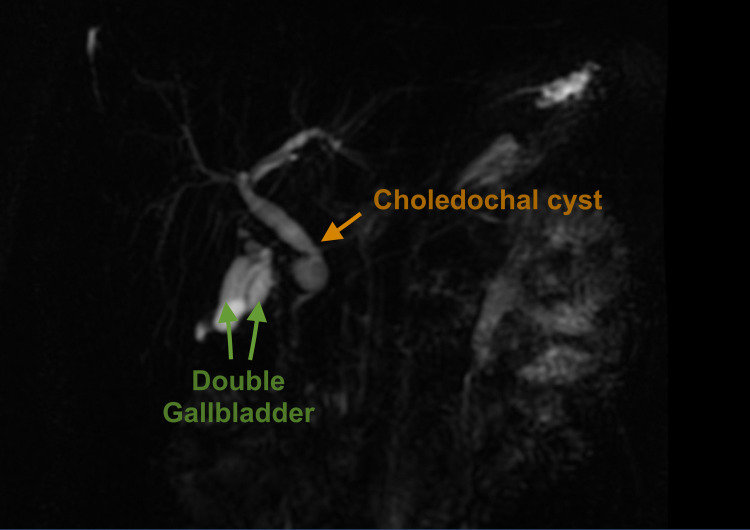
Magnetic resonance cholangiopancreatography showing double gallbladder and choledochal cyst.

A 26-year-old man presented to our clinic with recurrent right upper quadrant abdominal discomfort lasting one month accompanied by feelings of nausea without emesis. He had no yellowish discoloration of the skin and no abdominal swelling or fever on physical examination. The patient stated he was not constipated, nor does he have familial history of malignancy. The results of his laboratory workup, including complete blood count and liver function tests, were within reference ranges. An abdominal ultrasound revealed duplicate gallbladders. Magnetic resonance cholangiopancreatography (MRCP) confirmed the presence of a second gallbladder and a type I choledochal cyst (Figure 1).

We conducted an exploratory laparotomy which confirmed the radiology findings. We began the dissection by mobilizing the head of the pancreas. Blunt dissection and electrocautery were used to open the hepatic portal system, while the common bile duct was given a wide margin to avoid injuring the portal vein. We cleared the lateral, posterior, and medial attachments and mobilized the two gallbladders using electrocautery.

**Figure 2:**
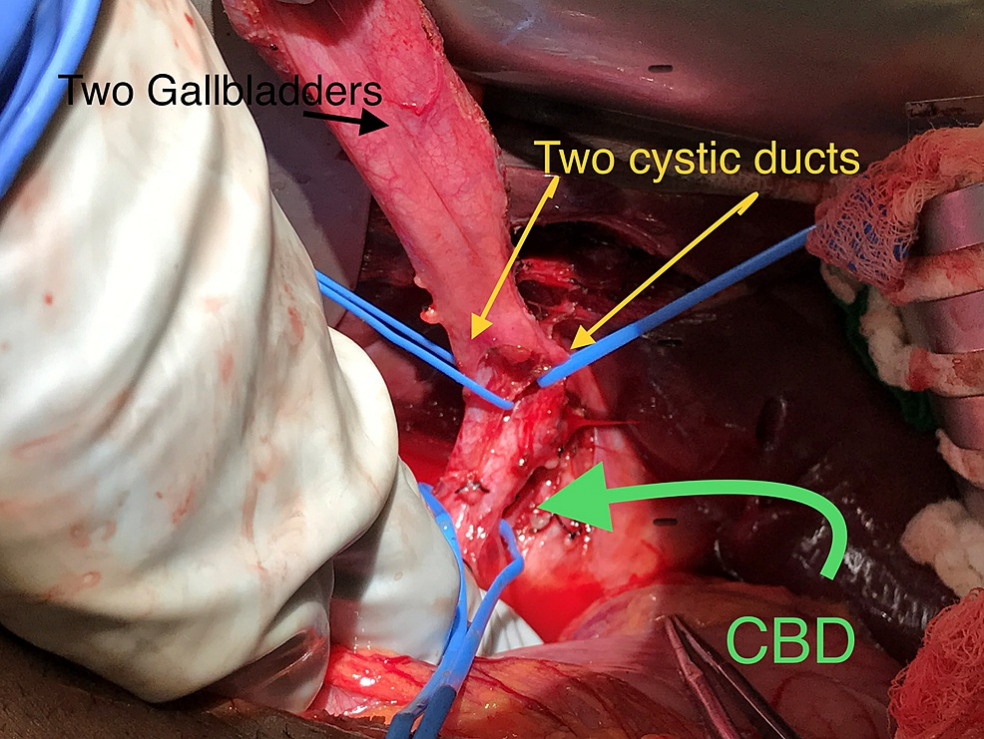
Surgical anatomy showing two gallbladders, two cystic ducts, and CBD. CBD: Common bile duct.

We mobilized the pancreas entirely using blunt and sharp dissection (Figure 2).

**Figure 3:**
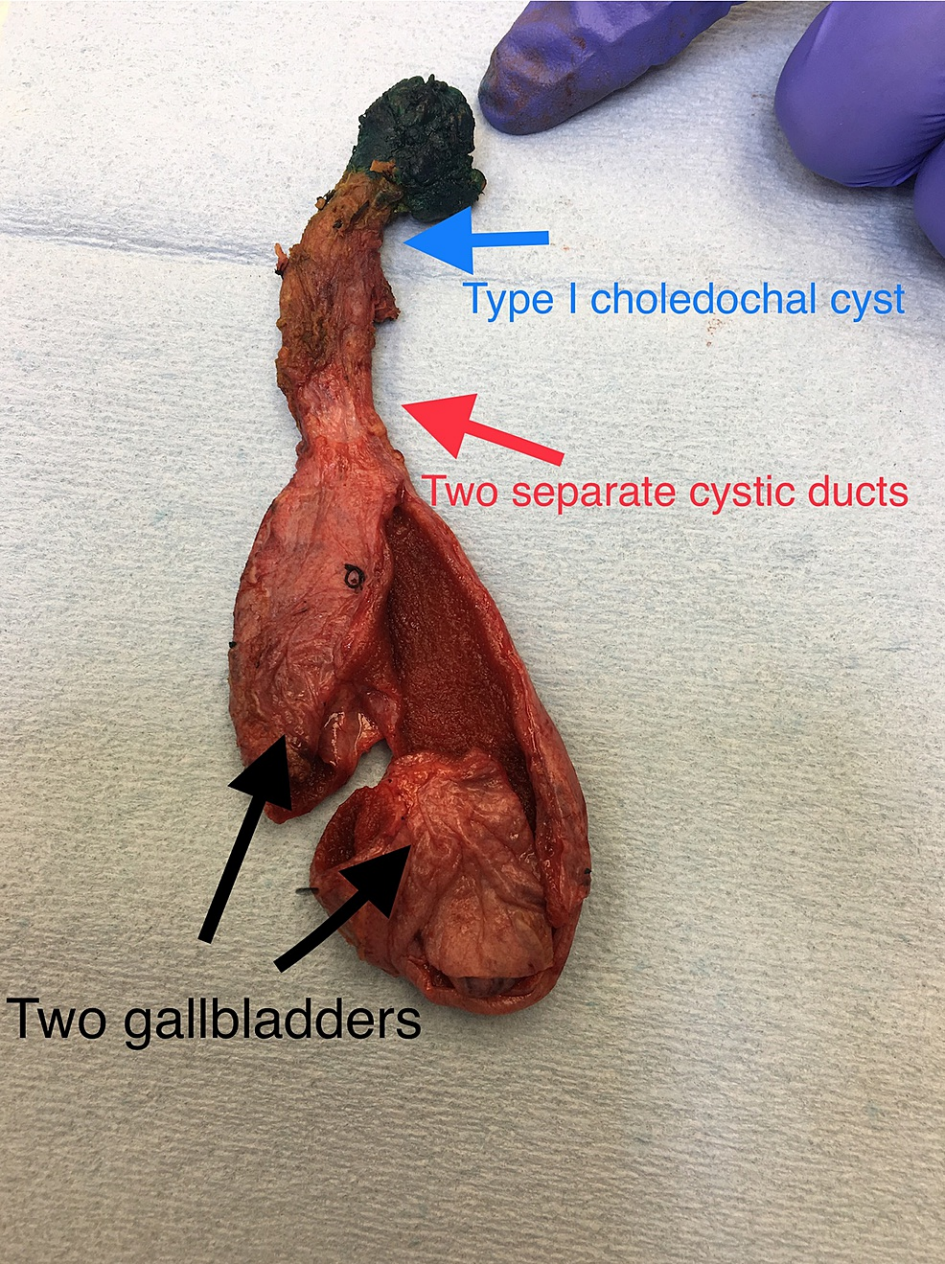
Gross sample presenting dual gallbladder, two cystic tubes, and type I choledochal cyst.

The large vessels were double clamped with the distal intrapancreatic joint bile tube. We tied and divided the distal joint bile duct 0.75 cm beneath the right and left hepatic tubes (Figure 3).

We created a jejunostomy after identifying a loop of jejunum, and we created a mesocolon window then conducted a hepaticojejunostomy. We placed a drain after confirming there were no leakages from the anastomosis. We sent samples for histopathological examinations, which revealed a choledochal cyst and a double non-communicating lumen lined by muscularis propria, columnar epithelium, and lamina propria adjoined to the adventitial layer. The patient recovered from the surgery with no complications and was doing well at his one-year postoperative follow-up.

## Discussion

Double gallbladder concurrent with a choledochal cyst is uncommon. Boyden classified the gallbladder’s association to the cystic duct into two main classes: split-primordium (doubled) or accessory gallbladder [3]. Harlaftis et al. modified the classification into two categories according to morphology and embryogenesis [4]. They defined type 1 as V-shaped and Y-shaped replicate gallbladders with a solitary duct connecting to the common bile duct. Type 2 indicated accessory gallbladders with more than two cystic ducts draining into the biliary tree separately, similar to our patient’s anatomy.

Choledochal cysts are categorized according to the degree of biliary tract dilation and location [5]. Type 1 cysts involve extrahepatic bile duct dilation and are the most common, appearing in 75% to 85% of cases, including our patient [5]. Researchers believe these cysts are congenital, but their exact etiology is unknown [5].

The clinical presentation of extra gallbladder concurrent with choledochal cysts can range from no symptoms to intermittent abdominal pain, jaundice, fever, or even cholangiocarcinoma. Double gallbladder does not present with any specific symptoms, does not increase disease possibility in either lobe, and is usually of little clinical significance unless disease is present. Diagnostic modalities start with obtaining transabdominal ultrasonography, given its high sensitivity for diagnosing gallstones, cholecystitis, or other anomalies. However, since double gallbladder can mimic other pathologies, further investigations may be required, including MRCP, computed tomography, cholangiography, percutaneous transhepatic cholangiography, or intraoperative cholangiogram to delineate the duct structures.

There is no role for prophylactic cholecystectomy in accidentally diagnosed double gallbladder. However, when it is symptomatic, the management of double gallbladder can be done laparoscopically or via open surgery [6].

## Conclusions

This case represents the first report of two relatively rare congenital conditions (i.e., double gallbladder and choledochal cyst) in the same patient. Double gallbladder is an unusual congenital condition that may mimic a choledochal cyst, even on an investigation such as MCRP. Surgeons should differentiate between the two abnormalities and remove the gallbladders when the patient is symptomatic. Later, they can perform an open or laparoscopic procedure, depending on the operating surgeon’s expertise. Knowledge of anatomy and a high level of alertness for congenital abnormalities is vital for safe surgical operation and optimal patient outcomes.
